# Impaired Mitochondrial Network Morphology and Reactive Oxygen Species Production in Fibroblasts from Parkinson’s Disease Patients

**DOI:** 10.3390/biomedicines12020282

**Published:** 2024-01-25

**Authors:** Kristina A. Kritskaya, Evgeniya I. Fedotova, Alexey V. Berezhnov

**Affiliations:** Institute of Cell Biophysics, Russian Academy of Sciences, Federal Research Center “Pushchino Scientific Center for Biological Research of the Russian Academy of Sciences”, 3 Institutskaya Street, 142290 Pushchino, Russia; delf-fenka@rambler.ru

**Keywords:** mitochondrial network, Parkinson’s disease, super-resolution microscopy, fibroblasts

## Abstract

The mitochondrial network (MN) is a dynamic structure undergoing constant remodeling in the cell. It is assumed that perturbations to the MN may be associated with various pathologies, including Parkinson’s disease (PD). Using automatic image analysis and super-resolution microscopy, we have assessed the MN parameters in fibroblasts from patients with established hereditary PD mutations (associated with PINK1, LRRK2, and α-synuclein, as well as PINK1 and Parkin proteins simultaneously) under normal conditions and after hydrogen peroxide-induced stress. Fibroblasts with the Pink1/Parkin mutation are most different in morphology to fibroblasts obtained from conditionally healthy donors: the MN is larger, and it contains longer mitochondria and accumulated individual mitochondria. In addition to MN, we evaluated other cellular parameters, such as cytosolic and mitochondrial ROS production and mitochondrial membrane potential. It has been shown that mitochondria of fibroblasts with mutations in genes encoding PINK1, α-synuclein, and Pink/Parkin tend towards hyperpolarization and cytosolic ROS overproduction, while mitochondrial ROS production was higher only in fibroblasts with PINK1 and α-synuclein mutations.

## 1. Introduction

**Figure 1:**
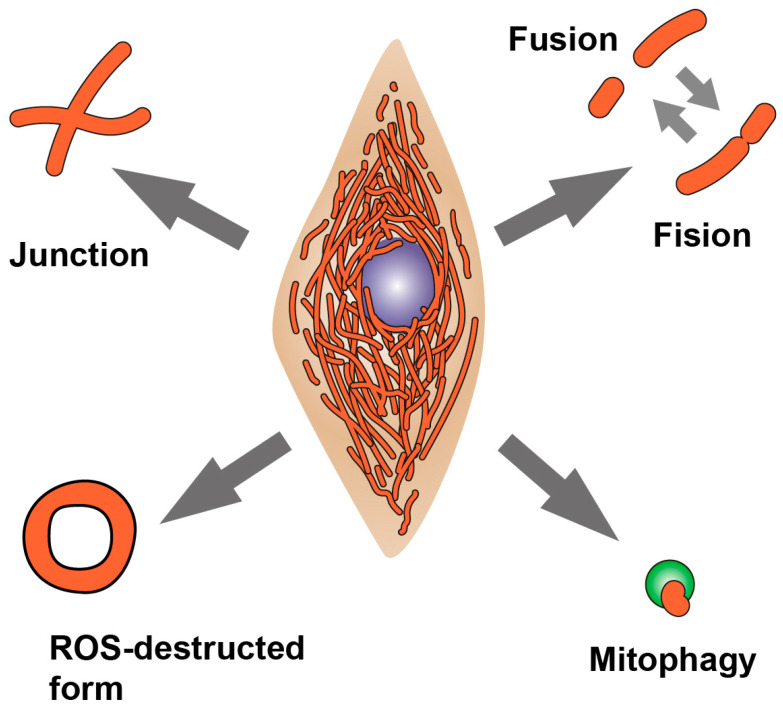
Processes involved in remodeling of the mitochondrial network morphology. The junction formation and mitochondria elongation occur by mitochondrial fusion, while separation of mitochondria from the network occurs by mitochondrial fission. Elimination of damaged mitochondria occurs through mitophagy and autophagy. In addition to these processes, mitochondrial morphology is also affected by oxidative stress, in which abnormal forms of mitochondria appear, such as “donuts” and “drops”.

Parkinson’s disease (PD) is a complex multifactorial neurodegenerative disease, including sporadic and hereditary forms accompanied by the development of mitochondrial dysfunction and oxidative stress [1]. Hereditary forms of PD are only a small proportion of all cases of PD, but studying them allows us to understand the fundamental mechanisms of this pathology. Since mitochondria perform a number of important functions in the cell—energy production, intracellular signaling, control of intracellular calcium homeostasis, triggering apoptosis, etc.—for the normal functioning of the cell, it is important to maintain functional healthy mitochondria and remove the damaged ones. To eliminate the toxic effects of dysfunctional mitochondria, organisms have developed a complex system of mitochondrial quality control implemented through the processes of mitochondrial fission/fusion [2] and the regulation of mitochondrial biogenesis and mitophagy. In the cell, mitochondria exist in the form of a dynamic structure—the mitochondrial network (MN), the morphology of which depends on the equilibrium of these opposite processes—as well as mitochondrial dynamics and autophagy (Figure 1).

Changing the MN morphology is also necessary for the regulation of metabolic processes, neuronal differentiation, and apoptosis [3]. Mitophagy is the process of selective autophagy of mitochondria by lysosomes. It is known that such proteins as PTEN-induced kinase 1 (PINK1) and Parkin RBR E3 ubiquitin protein ligase (Parkin) participate in mitophagy [4], and the disruption of their functions as a result of mutations leads to the development of hereditary PD [5,6].

### 1.1. Mitochondrial Fusion and Fission

Mitochondrial fusion and fission contribute to the adaptation of cells under various conditions, including oxidative stress [3]. With minor damage, individual mitochondria can merge with the MN to share resources, optimize respiration, and synthesize ATP. For instance, mitochondria with violated mitochondrial DNA (mtDNA) have been shown to fuse with intact mitochondria, allowing them to mix and efficiently redistribute mtDNA, as well as proteins and metabolites [7]. The process of mitochondrial fusion is realized due to the activity of GTPases that bind the outer mitochondrial membranes—Mitofusin-1 (Mfn1) and Mitofusin-2 (Mfn2)—and the inner one—Dynamin-like 120 kDa protein (OPA1). Disruption of fusion processes leads to the formation of a highly fragmented MN, which is characteristic of neurodegenerative diseases such as Charcot–Marie–Tooth disease and Dominant Optic Atrophy [8]. In the case of severe damage of individual mitochondria, their fusion with the MN can be dangerous for the entire MN; therefore, such mitochondria must be separated and safely degraded by mitophagy and autophagy. Mitochondrial fission is the opposite of fusion and is necessary, among other things, for the segregation of dysfunctional mitochondria. Mitochondrial fission is regulated by the GTPase-dynamics-related protein 1 (Drp1), as well as the Fission 1 (Fis1) and Mitochondrial fission factor (Mff) proteins [9]. When the fission processes are disrupted, the MN contains highly elongated mitochondria, which may also be associated with pathological processes. Thus, it was shown that in Purkinje cells, the absence of the main mitochondrial division protein, DRP1, first leads to the elongation of mitochondria, which then swell due to oxidative damage [10].

### 1.2. Mitochondrial Network Morphology and Diseases

Maintaining a functional MN is especially important for tissues that have a narrow specialization and high metabolic activity, such as neurons as well as cardiac and skeletal muscles. Since these cells are long-lived and postmitotic, they cannot divide damaged mitochondria between daughter cells and, unlike dividing cells, use more of the fusion/fission mechanism to preserve or restore the functions of damaged mitochondria [3]. At the moment, it is assumed that impairment in the morphology and dynamics of the MN play a significant role not only in Charcot–Marie–Tooth disease and Dominant Optic Atrophy but also in PD. For instance, it has been shown that mtDNA mutations accumulate because of MN disruption in PD dopaminergic neurons [11]. In addition, it has been reported that under the influence of various stress factors on the cell, the length of mitochondria decreases/increases, and structures resembling “donuts” (fully looped mitochondria) and “drops” (fragmented mitochondria) appear [12]. Assessing MN parameters such as length, connectivity, and the mitochondrial footprint may provide insight into the role of MN in pathology [12,13]. However, there is still no clear idea of how various mutations associated with PD can affect the MN morphology in other somatic cells and how this is related, in particular, to the level of oxidative stress. Fibroblasts are a convenient model for studying the MN morphology, since they are large sprawled cells.

In present work, using automatic image analysis and super resolution microscopy, we studied the parameters of the MN in fibroblasts from patients with established hereditary PD, associated with the following: (1) mutations in the PINK1 gene, encoding PTEN-induced kinase 1; (2) simultaneous mutations in genes PINK1 and PARK2, encoding PINK1 and Parkin RBR E3 ubiquitin protein ligase; (3) A53T point mutation in SNCA gene, encoding α-synuclein; (4) G2019S mutation in LRRK2 gene, encoding leucine-rich repeat kinase 2 protein. Moreover, we have analyzed cellular parameters of these fibroblast lines, such as the rate of mitochondrial and cytosolic ROS production as well as mitochondrial membrane potential under normal conditions and in response to stress.

## 2. Materials and Methods

### 2.1. Materials

H_2_DCFDA, MitoSOX Red, and MitoTracker Red CM-H_2_XRos were purchased from Invitrogen (Waltham, MA, USA); DMEM, FBS, Glutamax, Sodium Pyruvate, and TrypLE™ Express were purchased from Gibco, (Carlsbad, CA, USA); HBSS was purchased from PanEco (Moscow, Russia).

### 2.2. Cell Lines

All cell cultures were kindly provided by prof. Andrey Y. Abramov (UCL Institute of Neurology, London, UK). Cell cultures of human skin fibroblasts carrying mutations in the genes encoding alpha-synuclein (a-syn, A53T het), PINK1 (homozygous p.Try90Leufsx12), PINK1 and Parkin (PINK1/Parkin, PARK2 R275W/WT + PINK1 p.Try90Leu fs*12/WT), and the G2019S mutation in LRRK2 gene, as well as control lines of fibroblasts, were used as objects in this study (Table 1). We used the same cell lines as in the previous study [14].

Cells were cultured on 25 cm^2^ culture flasks in DMEM containing 10% FBS (Invitrogen, Waltham, MA, USA), 2 mM glutamine, and 1 mM pyruvate at 37 °C, 5% CO_2_, and 100% humidity. Upon reaching 80–85% confluence, the cells were split to maintain the culture or seeded on round 25 mm glass coverslips for the experiment. All cells used for experiments were not older than 18 passage.

### 2.3. Hydrogen Peroxide-Induced Stress

Hydrogen peroxide was added to the incubation culture medium at 37 degrees for 1 h, after which it was washed three times and left to rest for another hour, after which the experiment was started. The concentration of hydrogen peroxide was selected from the calculation of the dose leading to the death of 20% of cells for control fibroblast lines.

### 2.4. ROS Measurements

To assess cytosolic ROS production, the cells were loaded with the H_2_DCFDA probe (10 μM, 40 min), followed by washing with HBSS. Fluorescence registration was carried out using a Spark10M tablet reader (Tecan Group, Männedorf, Switzerland) or an imaging station based on a Leica DMI6000 B inverted microscope (Leica Microsystems, Wetzlar, Germany) using a standard FITC filter set (excitation: 494 ± 10 nm, emission: 535 ± 10 nm) with a 20× objective lens. Recording speed: 1 frame/5 s. To assess ROS production in mitochondria, cells were loaded with a MitoSOX Red probe (5 μM, 15 min) or MitoTracker Red CM-H_2_XRos (100 nM, 30 min), followed by washing with HBSS. Registration was performed using a standard Texas Red filter set (excitation: 575 ± 10 nm, registration: 624 ± 20 nm) and a 20× objective lens. Recording speed: 1 frame/5 s.

### 2.5. Mitochondrial Membrane Potential Measurements

The mitochondrial membrane potential (Δψm) was measured by incubating cells with 25 nM tetramethylrhodamine methyl ester (TMRM) fluorescent dye in a buffered saline solution (HBSS) for 40 min at room temperature. Fluorescent images were obtained using a Zeiss 900 CLSM (Carl Zeiss Microscopy GmbH, Jena, Germany) confocal microscope equipped with a ×63 oil immersion objective, and during the measurements, the TMRM remained in the HBSS solution. Illumination intensity was kept to a minimum (0.1–0.2% of laser output) to avoid phototoxicity. The cells were excited with a laser at 561 nm, and fluorescence was detected above 580 nm. FCCP, which is uncouplers of oxidative phosphorylation, were used to assess mitochondrial function. When FCCP is added, TMRM fluorescence is quenched. The mitochondrial membrane potential was estimated as the difference in TMRM fluorescence (maximum signal minus the signal with the addition of 2 μM FCCP) and was taken as 100% in control fibroblasts without treatment.

### 2.6. Mitochondrial Network Morphology Analysis

**Figure 2:**
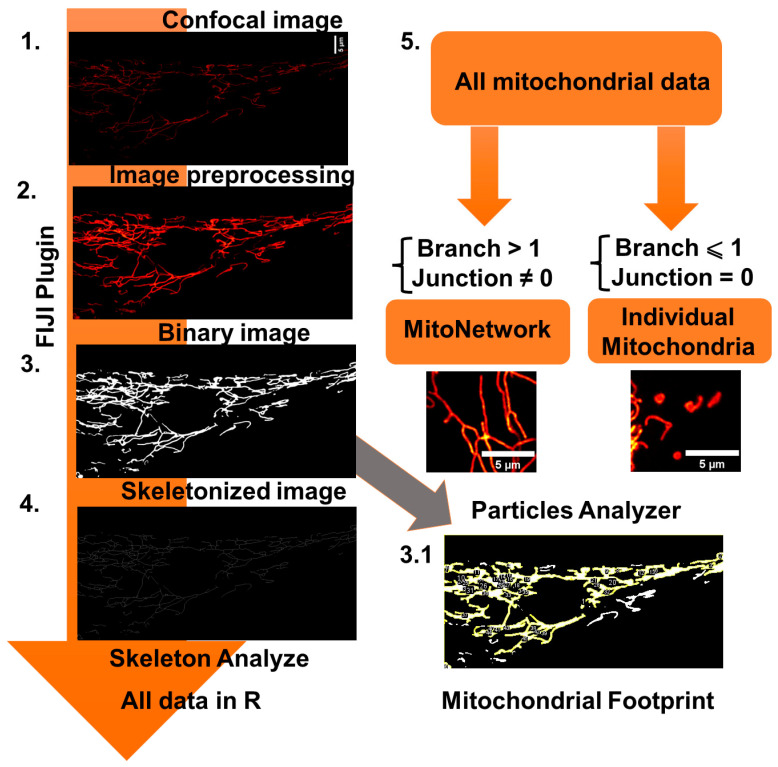
Mitochondrial network (MN) morphology analysis used in this work. (1.) Original confocal image was preprocessed (2.), which included background subtraction (rolling ball radius set on 50 pixels), median filter, and local contrast enhance (CLACHE) functions. (3.) Next, the image was binarized using threshold by the “Otsu” method. (3.1) Next, the image was copied, and the mitochondrial footprint was calculated by the sum of the mitochondrial areas in the “Particle analyzer” Fiji plugin. (4.) Furthermore, the image was skeletonized using the “Skeletonize” function and processed using the built-in “Skeleton Analysis” function. (5.) Next, the table of the received data was transmitted to the R programming environment. The data table was filtered using the “filter” function from “dplyr” package into two data subsets, “mitochondrial network” and “individual mitochondria”, according to the described scheme (mitochondrial networks were considered objects that consist of more than one branch and non-zero junction, whereas individual mitochondria consist of only one branch and zero junction).

To analyze the MN morphology, images of cells loaded with TMRM (25 nM), which constantly remained in the working solution, were taken using a Zeiss 900 CLSM (Carl Zeiss Microscopy GmbH, Jena, Germany) confocal microscope equipped with a ×63 oil immersion objective. Resolution is 23.4 pixels per micron. The mitochondrial area may depend on the cell volume. In order to take into account this effect on the MN area, we have previously estimated the volume of cells using Calcein AM fluorescence. Calcein at a concentration of 5 μM was loaded into cells for 30 min at room temperature, after which Z-stack images were obtained on a confocal microscope and the fluorescence area was calculated. It was found that there is no difference in this parameter in the studied cell lines. Next, we used a self-written Fiji plugin for automatic batch processing. The protocol for assessing the MN morphology was based on the approaches described earlier [12,15,16]. Detailed analysis steps are shown in Figure 2.

### 2.7. Statistical Analysis

Computer processing and image analysis of cell cultures were carried out using Fiji (https://imagej.net/software/fiji/downloads) and R-Studio (https://posit.co/download/rstudio-desktop/). OriginPro2018 was used for plotting. For analysis, two control lines of fibroblasts were combined. When processing data on the measurement of ROS production, curves were obtained, then were linearly approximated, and the rate of the fluorescent signal increase was calculated (control was taken as 100%).

Statistical analysis was performed using the “dplyr” package and OriginPro2018. Prior to hypothesis testing, all data were assessed for normality using the Shapiro–Wilk test. All measured variables were found to meet normality assumptions (*p* > 0.05). As a result, parametric tests were used for all statistical comparisons. Mitochondrial length values, at both the individual and network level, followed a non-normal distribution within single cell. Therefore, the median of the individual length measurements was calculated for each cell. These median values were normally distributed (Shapiro–Wilk test, *p* > 0.05) across the cell population. 

Differences between control and mutated fibroblasts were analyzed using one-way and two-way ANOVA (post Tukey test with Bonferroni correction). Statistical significance for cell lines before and after exposure to hydrogen peroxide was performed using an unpaired Student’s *t*-test (*p*-value < 0.05). Additionally, analysis of covariance (ANCOVA) was conducted with sex and age of fibroblast donors included as covariates, in order to determine their potential effects. The interaction effect between age and sex was not statistically significant (Supplementary Material, S1). The results were presented as box and whisker plots showing the median, mean, interquartile range, and total range.

## 3. Results

### 3.1. Mitochondrial Network Morphology of Fibroblasts

We investigated the MN morphology using an automatic plugin self-written in the Fiji and R programing languages. We evaluated five different parameters of MN: the total mitochondrial area (mitochondrial footprint) in µm^2^, the size of mitochondria in the MN, the ratio of the MN and individual mitochondria in cell, the size of individual mitochondria, and the connectivity of MN in fibroblasts with mutations from PD patients and conditionally healthy individuals under normal conditions and after H_2_O_2_-induced stress. As is known, hydrogen peroxide can increase the production of superoxide by mitochondria in a positive feedback loop according to the RIRR (ROS-Induced ROS Release) effect [17,18].

**Figure 3:**
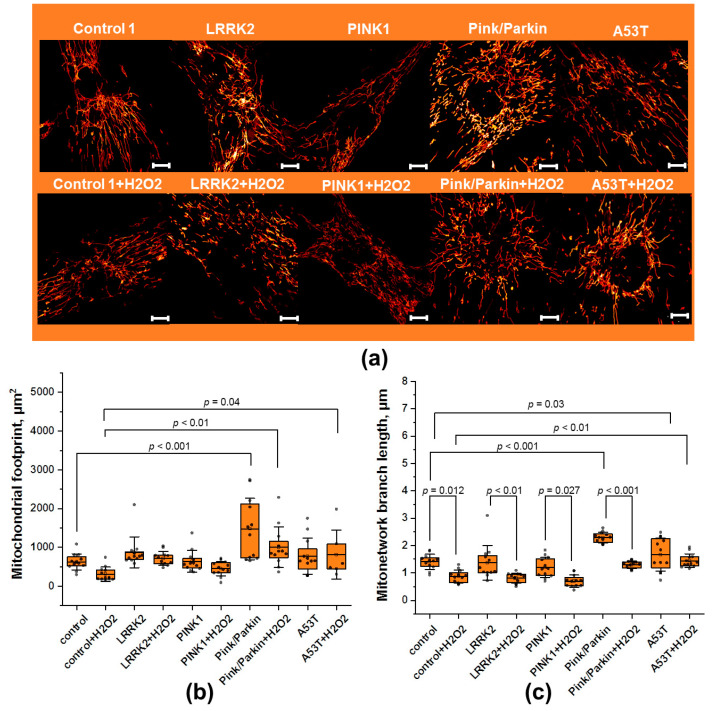
Mitochondrial network morphology in fibroblasts carrying PD-related mutations. (**a**) Representative microphotographs of fibroblasts stained with TMRM and obtained by a super-resolution confocal microscopy (applied Fiji “Red hot” LUT, scalebar 10 μm). (**b**) Mitochondrial footprint measured by TMRM fluorescence using the “particle analyzer” plugin in Fiji under normal conditions and after hydrogen peroxide (150 µM for 1 h) stress. (**c**) The mitochondrial branch length in the network (MN) measured using the “skeleton analyzer” plugin in Fiji under normal conditions and after H_2_O_2_-induced stress (150 µM for 1 h). *n* = 12 cells in 3 independent experiments. Box plots show median and interquartile range, and dots represent individual data points. Statistical significance for control and mutated fibroblasts was determined using one-way ANOVA with Tukey’s multiple comparison test. The Bonferroni correction was used to adjust the significance level for multiple comparisons (adjusted *p*-value = 0.005). Statistical significance for cell lines before and after exposure to peroxide was performed using an unpaired Student’s *t*-test (*p*-value < 0.05).

We estimated the mitochondrial footprint using the total fluorescence area of TMRM (Figure 3a). Staining mitochondria with potential-dependent TMRM gave confidence that it only stained energized mitochondria. A preliminary assessment of the cell volume was carried out using Calcein AM fluorescence, and no difference was found in the cell volume of the studied cell lines. The mitochondrial footprint (Figure 3b) in all fibroblasts, except Pink/Parkin, does not significantly differ from the control (for control it is 625 ± 196 µm^2^; for LRRK2—873 ± 399 µm^2^; PINK1—639 ± 279 µm^2^; A53T 765 ± 468 µm^2^). The Pink/Parkin mitochondrial footprint is significantly higher by 2.3 times that of the control, at 1474 ± 793 µm^2^. When exposed to hydrogen peroxide, the mitochondrial footprint across all fibroblasts tends to decrease. However, in Pink/Parkin and A53T fibroblasts, it remains significantly higher than in control cells (by 3.2 and 2.6 times, respectively).

Next, we evaluated the length of mitochondria (Figure 3c) in the MN (mitochondrial branch length). The mitochondrial branch length can be an important parameter showing the adaptation of the cell to substrate deprivation and stress, and it may change in pathology. As mentioned above, an MN is considered to be a structure containing strictly more than one branch and a non-zero connection. The mitochondrial branch lengths were higher in fibroblasts with Pink/Parkin (2.28 ± 0.19 μm) and A53T (1.66 ± 0.6 μm) mutations compared to the control (1.41 ± 0.27 μm) as well as other mutated fibroblasts (LRRK2—1.37 ± 0.63 μm; PINK1—1.19 ± 0.36 μm). As expected, hydrogen peroxide addition activated mitochondrial fission, leading to a significant decrease in mitochondrial branch length across almost all fibroblasts except A53T. The coefficients of mitochondrial length reduction are as follows: control—1.60; LRRK2—1.65; PINK1—1.70; Pink/Parkin—1.79.

**Figure 4:**
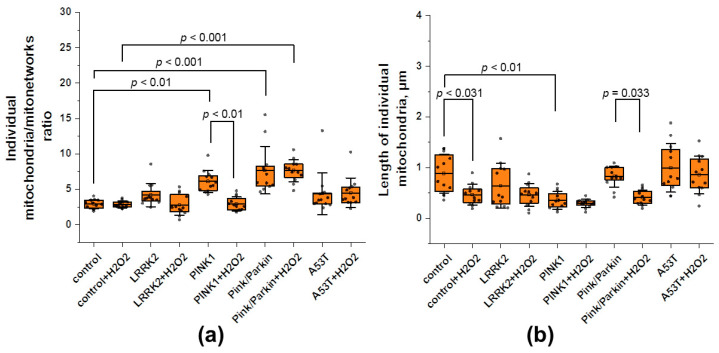
The ratio and length of individual mitochondria in fibroblasts of patients with Parkinson’s disease and established mutations and healthy individuals. (**a**) The ratio of individual mitochondria to mitochondrial networks (MNs) under normal conditions and after hydrogen peroxide (150 µM for 1 h) stress. MN is considered to be a structure containing strictly more than one branch and a non-zero connection, whereas individual mitochondria contain only one branch and zero junction. (**b**) The median length of individual mitochondria before and after treatment with hydrogen peroxide (150 µM for 1 h) stress. *n* = 12 cells in 3 independent experiments. Box plots show median and interquartile range, and dots represent individual data points. Statistical significance for control and mutated fibroblasts was determined using one-way ANOVA with Tukey’s multiple comparison test. The Bonferroni correction was used to adjust the significance level for multiple comparisons (adjusted *p*-value = 0.005). Statistical significance for cell lines before and after exposure to peroxide was performed using an unpaired Student’s *t*-test (*p*-value < 0.05).

The accumulation of individual mitochondria may indicate an imbalance of fission/fusion, as well as mitophagy. Interestingly, early reports suggest that the mitophagy level in these mutated fibroblasts is comparable with conditionally healthy controls [14]. In addition, since TMRM dye stains mitochondria with mitochondrial membrane potential, we believe that in our case it is possible to analyze fission/fusion balance. Figure 4a shows the ratio of individual mitochondria to the MN, since the absolute values of individual mitochondria vary in a wide range, even in the same culture analyzed. Thus, the greater this value, the more individual mitochondria in the cell. Under normal conditions, the ratio of individual mitochondria for fibroblasts with PINK1 (6.1 ± 1.5) and Pink/Parkin (7.6 ± 3.3) mutations are significantly higher by 2.1 and 2.6 times, respectively, than in control cells (2.9 ± 0.6). For fibroblast with mutations LRRK2 it is 4.1 ± 1.6, for A53T it is 4.3 ± 2.9, and after hydrogen peroxide stress it is 2.7 ± 1.1 and 4.5 ± 2.8, respectively. Interestingly, after hydrogen peroxide stress, the ratio of individual mitochondria of PINK1 cells significantly decreases by 2.1 times, whereas for the Pink/Parkin mutation, on the contrary, it does not change and remains significantly higher than in the control.

Next, we estimated the median length of individual mitochondria (Figure 4b), since we were interested in which mitochondria make up the population of non-MN mitochondria in cells with various mutations. We assumed that the length of individual mitochondria should be less than mitochondrial branch in the MN. Indeed, the length of individual mitochondria for all the studied lines was lower than the network ones (control—0.88 ± 0.38; LRRK2—0.64 ± 0.44; PINK1—0.37 ± 0.17; Pink/Parkin—0.82 ± 0.2; A53T—0.99 ± 0.47). At the same time, in cells with the PINK1 mutation, it is significantly lower by 2.3 times than in control cells. After the hydrogen peroxide addition, there is a tendency to individual mitochondrial length decrease for all studied fibroblasts; however, this parameter significantly decreases only in control cells (by 1.9 times) and Pink/Parkin cells (by 2 times), possibly due to higher initial values.

**Figure 5:**
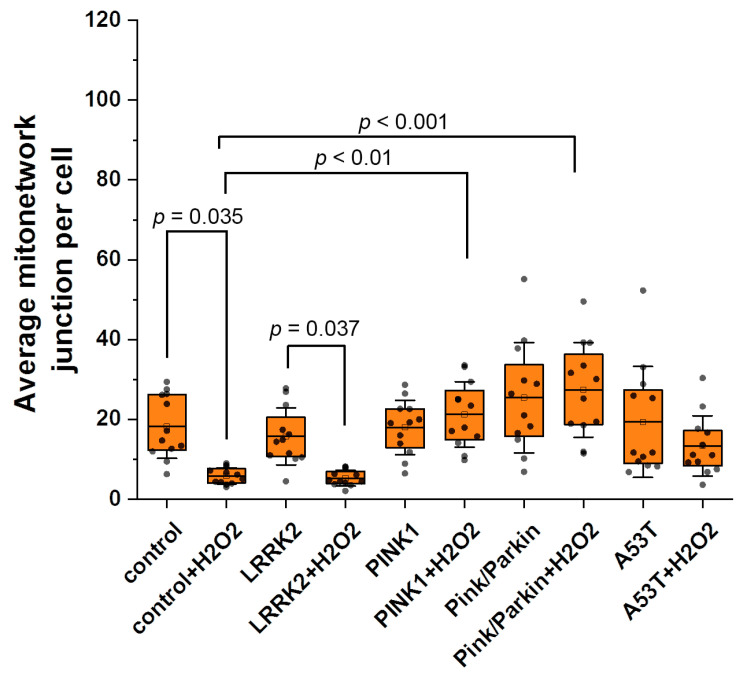
The average number of junctions in mitochondrial networks (MN connectivity) in fibroblasts of patients with Parkinson’s disease and established mutations and healthy individuals. The MN connectivity was evaluated under normal conditions and after hydrogen peroxide-induced stress (150 µM, 1 h). *n* = 12 cells in 3 independent experiments. Box plots show median and interquartile range, dots represent individual data points. Statistical significance for control and mutated fibroblasts was determined using one-way ANOVA with Tukey’s multiple comparison test. The Bonferroni correction was used to adjust the significance level for multiple comparisons (adjusted *p*-value = 0.005). Statistical significance for cell lines before and after exposure to peroxide was performed using an unpaired Student’s *t*-test (*p*-value < 0.05).

Furthermore, we evaluated the MN connectivity (Figure 5) by calculating the average number of junctions in the MN per cell. A change in mitochondrial connectivity may indicate cell adaptation to decreased substrate supply, a violation of bioenergetics and mitochondrial fission/fusion. Under normal conditions, the average number of junctions in the MN for all cell lines does not differ significantly (control—18.2 ± 7.5; LRRK2—15.6 ± 7.1; PINK1—17.9 ± 6.8; A53T—19.3 ± 13.9; Pink/Parkin—25.4 ± 13.8). When hydrogen peroxide is added, the average number of junctions in the MN decreases for control cells and fibroblasts with LRRK2 mutation significantly (the reduction coefficient for control is 3.13; for LRRK2—2.1), whereas in cells with PINK1, Pink/Parkin, and A53T mutations, connectivity does not change and is significantly higher than in control cells (for PINK1 by 3.7 times and Pink/Parkin by 4.6 times).

Thus, we investigated five different parameters describing MN morphology in fibroblasts with PD-associated mutations. The greatest difference in the MN parameters from the control was found for cells with a double mutation associated with mitophagy: Pink/Parkin.

### 3.2. Mitochondrial Membrane Potential

**Figure 6:**
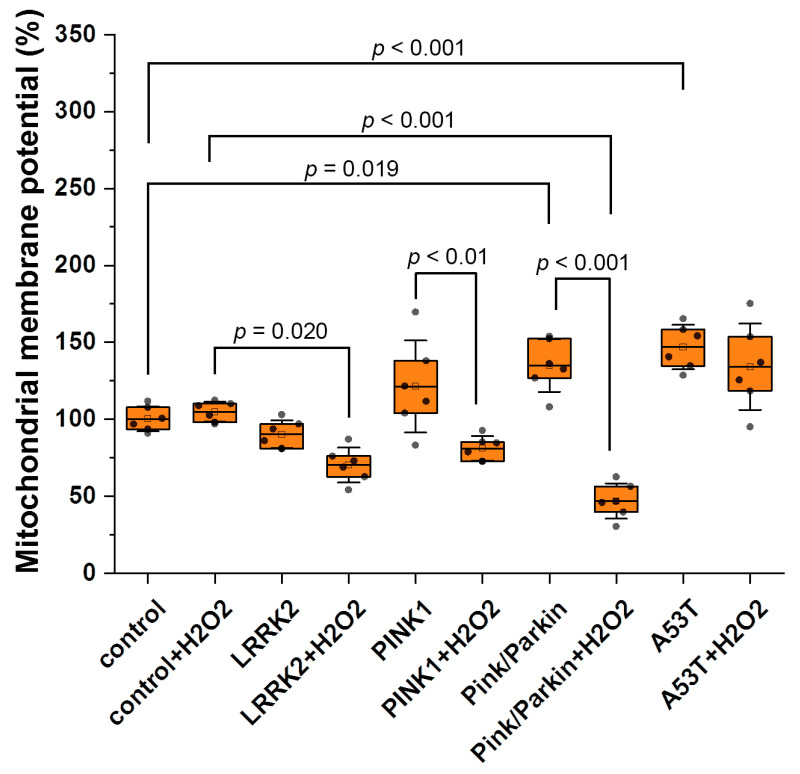
Mitochondrial membrane potential in control fibroblasts and in cells with PD-related mutations. The mitochondrial membrane potential was evaluated under normal conditions and after hydrogen peroxide-induced stress (50 µM, 1 h) as a percentage of the control TMRM fluorescence. Two controls were combined, and percentages were calculated from the mean of the two control cell lines. *n* = 60 cells in 3 independent experiments. Box plots show median and interquartile range, and dots represent the average mitochondrial potential from 10 cells. Statistical significance for control and mutated fibroblasts was determined using one-way ANOVA with Tukey’s multiple comparison test. The Bonferroni correction was used to adjust the significance level for multiple comparisons (adjusted *p*-value = 0.005). Statistical significance for cell lines before and after exposure to peroxide was performed using an unpaired Student’s *t*-test (*p*-value < 0.05).

The maintenance of the mitochondrial potential is necessary for efficient ATP synthesis, while changes in the mitochondrial membrane potential may indicate possible impairment of mitochondrial function. We evaluated the values of the mitochondrial potential using a potential-sensitive probe, TMRM (Figure 6). It was found that the mitochondrial membrane is significantly hyperpolarized in fibroblasts with Pink/Parkin (137.6 ± 21.2%) and A53T (154.5 ± 10.4%) mutations relative to the control (100%). After hydrogen peroxide-induced stress (150 μM, 30 min), there is significant decrease in mitochondrial membrane potential in fibroblasts with PINK1 (by 34.8 ± 8.3) and Pink/Parkin (by 90.7 ± 7.5) mutations, while in cells with the A53T mutation, the mitochondrial membrane potential remains hyperpolarized.

### 3.3. Cytosolic and Mitochondrial ROS Production Rate

**Figure 7:**
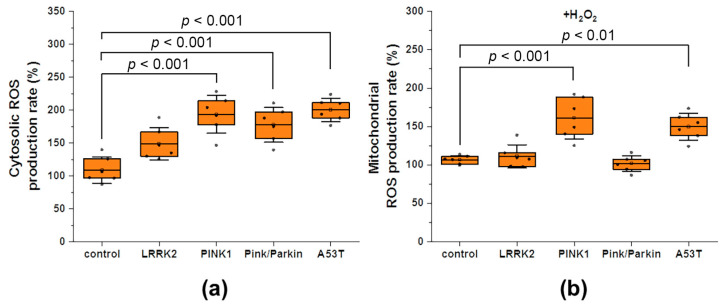
ROS production in controls fibroblasts and in fibroblasts with Parkinson’s disease-related mutations. (**a**) Cytosolic ROS production rate estimated by the time kinetics of DCF fluorescence (chemically reduced and acetylated forms of 2′,7′-dichlorofluorescein H_2_DCFDA probe) as a percentage of the control. (**b**) Mitochondrial ROS production rate estimated by the time kinetics of MitoSOX Red and MitoTracker Red CM-H_2_XRos probes (their fluorescent derivatives after oxidation) after hydrogen peroxide-induced stress (50 µM, 1 h) as a percentage of the control. Two controls are combined, and percentages are calculated from the mean of the two control cell lines. *n* = 60 cells in 3 independent experiments. Box plots show median and interquartile range, and dots represent the average cytosolic or mitochondrial ROS production rate from 10 cells. Statistical significance was determined using one-way ANOVA with Tukey’s multiple comparison test. The Bonferroni correction was used to adjust the significance level for multiple comparisons (adjusted *p*-value = 0.0083).

We measured the rate of cytosolic ROS production under normal conditions using the time kinetics of DCF fluorescence (chemically reduced and acetylated forms of 2′,7′-dichlorofluorescein H_2_DCFDA probe) and calculated the rate as a differential (Figure 7a). A significant increase in the rate of ROS production was found almost for all fibroblasts with mutations: PINK1 by 1.9 times (191.5 ± 36.6%), Pink/Parkin by 1.8 times (180.3 ± 31.1%), and 1.9 times (199.4 ± 21.4%) in A53T relative to the control taken as 100% (with the exception of the LRRK2 mutation fibroblast, where only a trend was observed). 

In addition to mitochondrial membrane potential, an important indicator of mitochondrial function is the rate of mitochondrial ROS production. Since we did not find any difference under normal conditions, we assumed that exogenous stress could reveal a change in the rate of mitochondrial ROS production in the studied fibroblasts. The rate of mitochondrial ROS production (Figure 7b) was measured after hydrogen peroxide addition (50 µM, for 1 h) by the time kinetics of MitoSOX Red and MitoTracker Red CM-H_2_XRos probes (their fluorescent derivatives after oxidation). After the addition of hydrogen peroxide, a significant increase in the rate of mitochondrial production was found for PINK1 fibroblasts (by 1.6 times—163.5 ± 32.1%) and A53T (by 1.4 times—147.6 ± 21.3%) relative to the control taken as 100%.

## 4. Discussion

This work is a quantitative analysis of the mitochondrial network (MN) morphology (parameters such as mitochondrial footprint, length of mitochondria in NW, the ratio of individual mitochondria, and their length, as well as mitochondrial connectivity) in fibroblasts obtained from patients with hereditary forms of PD and established mutations and also their response to hydrogen peroxide-induced stress. Two fibroblast lines studied in this work are associated with malfunctions in the mitophagy-related genes PINK1 and PARK2, and one line contains a mutation in a Leucine-rich repeat kinase 2 (LRRK2), a protein involved in autophagy. Another fibroblast line contains a point mutation in the gene encoding α-synuclein (A53T, SNCA), the aggregates of which are found in the brains of PD patients. As a control, we used fibroblasts from conditionally healthy individuals comparable in age to patients with PD, between which, in most cases, there were no significant differences and which were presented in combined form. In addition to the MN morphology, we evaluated the mitochondrial membrane potential and the rate of cytosolic and mitochondrial ROS production in these fibroblasts. A change in the stress response may indicate pathology. In our study, hydrogen peroxide was used as a stress model, since it is known that it causes ROS-dependent ROS production, and in some cases [17], it has revealed differences in the cell parameters that were not present under normal conditions. It is believed that MN morphology may depend on the external stress on the cell, substrate deprivation, etc., and the rearrangement of MN morphology is the most important for cell adaptation under various conditions [3,10,18].

The MN may be a potential target for the therapy of neurodegenerative diseases, including PD [19]. It is shown that with aging, mitochondria undergo fragmentation and lose area [20], which also may be associated with the development of neurodegeneration [21]. However, in this work we showed a significant change in the mitochondrial footprint only for the Pink/Parkin mutation fibroblasts, which, on the contrary, led to an increase in this area of the MN (Figure 3b), probably due to several synergistic reasons. Firstly, as it has been shown, the PINK1 and Parkin proteins are involved in the regulation of Drp1 activity through phosphorylation (increasing its activity) [22] and of Mitofusin activity through ubiquitination (inhibiting their activity) [23]. Accordingly, with aberrant function of the PINK1 and Parkin proteins, on the one hand, fission is disrupted, and on the other hand, fusion is not inhibited. In addition, since PINK1 and Parkin are key regulators of PINK1/Parkin-mediated mitophagy [24], aberrant function of these proteins may lead to decreased efficiency of mitochondrial degradation, and mitochondria may accumulate in the cell, contributing to the mitochondrial footprint. Interestingly, when exposed to stress, there was indeed a decrease in the mitochondrial footprint of fibroblasts, but for the most part it was not significant, and the mitochondrial footprint in Pink/Parkin mutation fibroblasts remained the largest. 

The change in the mitochondrial length may occur due to increased fusion or the disruption of fission. An increase in the mitochondrial length may indicate, for instance, an intensification of ATP production processes [25]. Conversely, oxidative stress has been shown to promote the activation of mitochondrial fission through post-translational modification and increased expression of Drp1 and Fis1, which may result in decreased mitochondrial length [26]. In our work, we found an increase in the median mitochondrial length of MN (mitochondrial branch) in cells with Pink/Parkin and A53T mutations (Figure 3c). Since Mfn1 and Mfn2 are involved in mitochondrial fusion and are known to be substrates for Parkin ubiquitination [27], it is not surprising that impaired Parkin function leads to an increase in the length of mitochondria due intense fusion. Interestingly, although it was previously shown that the expression of α-synuclein with A53T mutation in mammalian cells leads to fragmentation of mitochondria [28], we found the opposite effect. At the same time, it is worth noting that the peroxide-induced stress led to a significant decrease in the mitochondrial length in all the studied cells, except for fibroblasts with the A53T mutation. α-synuclein is expressed not only in neurons but also in fibroblasts [29], and its mutant form A53T has a greater ability to form pathological aggregates [30]. Since a number of previous studies have shown that α-synuclein and its mutant form can interact with the main fission proteins (Drp1, Fis1), blocking their function [31], as well as with cytoskeletal proteins, disrupting the process of mitochondrial fission [32], we believe that the increase in mitochondrial length in fibroblasts with the A53T mutation under normal and stress conditions may be associated with impaired mitochondrial fission due to the influence of the mutant α-synuclein form.

The accumulation of individual mitochondria may also indicate a violation of mitochondrial dynamics and mitophagy [15,16]. We found that for fibroblasts with Pink/Parkin and PINK1 mutations, the ratio of individual mitochondria to network ones is higher compared to the control (Figure 4a), which may indicate increased division or/and inefficiency of mitophagy. Interestingly, despite mutations in Parkin and PINK1 involved in the recruitment of mitochondria into mitophagy [15,33,34], early reports of these cells with mutations showed the levels of mitophagy in fibroblasts to be similar to control cells, studied by the double-staining of lysosomes and mitochondria [14]. Perhaps, from our results, it can be concluded that when analyzing mitophagy, it is necessary to look not only at the colocalized lysosomes and mitochondria but also the ratio of individual mitochondria in cells. It is noteworthy that under the action of hydrogen peroxide, the ratio of individual mitochondria to mitochondrial networks in fibroblasts with the PINK1 cell mutation decreased, whereas for the double Pink/Parkin mutation, it did not change. A decrease in the ratio of individual mitochondria may be associated with the activation of transcription factor, which is known to be involved in the launch of mitophagy and is dependent on stress [35,36,37,38]. Perhaps such stress can cause mitophagy activation, but only in the case of a single PINK1 mutation. This is consistent with data where it was previously shown that mild and sustained hydrogen peroxide (H_2_O_2_) stimulation induces Parkin-dependent mitophagy accompanied by downregulation of the mitophagy-associated proteins OPTN, NDP52, and MFN2 [39]. This protective effect of moderate stress during exercise or starvation may also play a role in increasing mitochondrial biogenesis via PGC-1α and DRP1 [25,39,40].

In addition to the ratio of individual mitochondria, we made an attempt to study the representative size of individual mitochondria in fibroblasts. To do this, we estimated the median length of individual mitochondria (Figure 4b). It was found that in control fibroblasts and fibroblasts with mutations, individual mitochondria are predominantly up to 1 µm in length. Surprisingly, the PINK1 mutation is characterized by shorter individual mitochondria, while the A53T mutation, on the contrary, is longer compared to the control. We hypothesized that the increase in the ratio of individual mitochondria of smaller sizes in the PINK1 mutant line occurs due to impaired mitophagy and the accumulation of damaged individual mitochondria, the length of which is shorter than that of “healthy mitochondria” (due to swelling of the matrix and their acquiring a spherical shape). To confirm this assumption, we additionally analyzed the aspect ratio of individual mitochondria in the PINK1 mutant line (lower aspect ratio values may indicate more “spherical” mitochondria). And, indeed, the aspect ratio of individual mitochondria both before and after treatment with hydrogen peroxide in the PINK1 mutant line is significantly lower than in control cells, which confirms our assumption (Figure S1 in the Supplementary Materials, S2). The impact of stress had an ambiguous effect on the size of individual mitochondria: in the control and in cells with the Pink/Parkin mutation, they became shorter, although there were no significant changes in other fibroblasts.

MN connectivity can also change in pathology and as a response to physiological conditions, for example, starvation [16]. We have shown that MN connectivity does not differ in fibroblasts with mutations and control cells under normal conditions (Figure 5). Interestingly, after hydrogen peroxide addition, the number of junctions in the MN of control fibroblasts and fibroblasts with the LRRK2 mutation significantly decreases, while for PINK1, A53T, and Pink/Parkin fibroblasts, it does not significantly change in response to stress. Possibly, this may be due to a violation in these cells of the mitochondrial fission machinery, because fission requires the coordinated work of the GTPase separating the inner and outer mitochondrial membranes [41]. When only the inner membrane separates, the mitochondria can still be connected to the rest of the MN [13].

According to modern concepts, mitochondrial dysfunction is a key factor in the pathogenesis of hereditary and sporadic PD. Mitochondrial function can be indicated by the potential of the mitochondrial membrane. We evaluated this parameter by TMRM fluorescence (Figure 6). It was found that fibroblasts with Pink/Parkin and A53T mutations are characterized by a significant increase in the hyperpolarization of the mitochondrial membrane, and such a trend is observed for the PINK1 mutation. Hyperpolarization of the mitochondrial membrane has been shown for aging cells and may be associated with oxidative stress and also as a consequence of increased energy requirements, substrate deprivation, or disruption of the ETC [42,43]. Interestingly, the cells with hyperpolarized membranes turned out to be sensitive to stress—in the control lines, the addition of hydrogen peroxide did not lead to any significant differences, whereas for A53T and Pink/Parkin and PINK1 cells, a significant decrease in mitochondrial potential occurred. 

Oxidative stress is a hallmark of neurodegenerative diseases; in addition, mitochondrial dysfunction also causes oxidative stress [1]. There is a complex interplay between oxidative stress and autophagy/mitophagy in cells: basal autophagy/mitophagy promotes segregation of oxidized proteins and damaged mitochondria, reducing oxidative stress. Defects in these pathways lead to the accumulation of dysfunctional mitochondria and ROS production [44]. On the other hand, moderate levels of ROS can induce autophagy/mitophagy through the activation of signaling proteins like AMPK and sirtuins, which stimulate the elimination of damaged mitochondria and misfolded proteins [45]. However, excessive oxidative stress inhibits autophagy: ROS overproductions causes damage to lysosomes, impairs autophagosome–lysosome fusion and suppresses the activation of genes regulating autophagy by inactivating transcription factors, while impaired autophagy in turn reduces the clearance of mitochondria producing ROS. This creates a vicious cycle, further increasing cellular damage [46]. Though it is believed that oxidative stress in PD is characteristic of brain neurons, we found an increase in the rate of cytosolic ROS production in almost all fibroblasts with mutations (Figure 7a). Despite the limitation that we cannot estimate the contribution of any particular form of ROS, using mitochondrial-oriented probes, it is possible to measure whether mitochondria are sources of ROS overproduction in fibroblasts with mutations. Since, under normal conditions, it was not possible to detect a difference in mitochondrial ROS production between control cells and fibroblasts with mutations, we applied a model with peroxide-induced stress (Figure 7b). After the hydrogen peroxide addition, a significant increase in mitochondrial ROS production was detected in fibroblasts with PINK1 and A53T mutations, which may also indicate ETC disruption and mitochondrial dysfunction [1]. It should also be noted that we did not find an increase in mitochondrial ROS production in fibroblasts with the Pink/Parkin mutation, possibly due to a decrease in mitochondrial potential after stress, which can disrupt the accumulation of fluorescent probes near the mitochondrial membrane.

To summarize, in fibroblasts from patients with PD and established mutations, we found various changes in MN morphology compared to control cells from conditionally healthy donors. Fibroblasts with a double Pink/Parkin mutation have the most different morphology, as their MN is larger and contains longer mitochondria. Despite the fact that the A53T mutation in the SNCA gene is not directly related to mitochondrial dynamics, the fibroblasts that contain it also showed significant differences in MN morphology, similar to Pink/Parkin cells. It is worth noting that a new parameter was introduced in this work, the ratio of individual mitochondria and MN, using which the accumulation of individual mitochondria in cells with the PINK1 and Pink/Parkin mutations was revealed. Remodeling of the MN in response to stress also differed in conditionally healthy control cells and fibroblasts with mutations, and to the greatest extent in cells with a double Pink/Parkin mutation.

It is important to note that the morphology of the mitochondrial network does not always correlate with function but may be a consequence of cell adaptation to conditions. In addition, it is necessary to investigate not only the MN morphology but also its relationship with metabolic signatures and the state of the cell [47]. Impairment in the mitochondrial membrane potential and the rate of mitochondrial and cytosolic ROS production were also estimated in this work, but we have not established a connection, whether this is the cause or consequence of changes in the MN morphology in these fibroblasts. 

Surprisingly, cells with the c mutation in the gene encoding LRRK2 kinase showed no significant differences in the studied parameters, although it is believed that this protein is also associated with autophagy and mitochondrial function [48,49].

We believe that such violations in fibroblasts with mutations do not have critical consequences as in neurons, since fibroblasts are relatively rapidly dividing cells, whereas neurons live throughout the life of an individual. However, as we have shown, these cells can be a promising and convenient model for studying the relationship between MN morphology and pathology associated with neurodegeneration. Furthermore, recent articles have shown that the MN can be a target for regulation of stem cell differentiation and for relieving various pathological conditions [50,51]. This is of great interest in light of the fact that neurons can be obtained from the fibroblasts of patients with PD to replace dead cells in the brain.

In addition to assessing the contribution of PD-associated mutations, we analyzed the effects of sex and age of fibroblast donors on MN changes using covariate analysis (Supplementary Material, S1). Although these parameters did not have significant effects in our data, a major limitation of this study is interindividual variability, which may introduce uncertainty in MN measurements. Despite the presence of high interindividual variability, the differences in mitochondrial parameters between the individual experimental groups and the controls were statistically significant. For example, the length of mitochondrial branches in Pink/Parkin fibroblasts was higher compared to controls, with *p*-value < 0.001. 

As data accumulate on how PD-linked mutations impact mitochondrial morphology, retrospective analyses may provide more conclusive insights. Overall, further studies with larger patient cohorts are needed to clarify the phenotypic consequences of specific mutations while accounting for intrinsic variability across individuals.

## 5. Conclusions

Thus, the present work can be useful for understanding the role of MN morphology in the development of the hereditary PD form by the example of fibroblasts from patients with an established disease.

## Figures and Tables

**Table 1:** Cell lines used in the study.

Fibroblasts Cell Line	Mutation	Diagnosis	Age	Sex
Control 1	–	Healthy donor	56	Male
Control 2	–	Healthy donor	49	Female
LRRK2	G2019S	PD	55	Male
PINK1	Homozygous p.Try90Leufsx12 in PINK1	PD	52	Female
PINK1/Parkin	Park2/Pink1 double heterozygous PARK2 R275W/WT + PINK1 p.Try90Leu fs*12/WT	PD	72	Male
A53T	A53T het in SNCA	Severe PD	52	Female

## Data Availability

The data supporting this study’s findings are available from the corresponding author upon reasonable request.

## Supplementary Materials

The following supporting information can be downloaded at: https://www.mdpi.com/article/10.3390/biomedicines12020282/s1, Supplementary S1. Evaluation of the influence of age and gender on the dependent variables; Table S1: Summary table of measured parameters; Figure S1: Aspect ratio of individual mitochondria in fibroblasts.
