# Supplementary material for: Impaired Mitochondrial Network Morphology and Reactive Oxygen Species Production in Fibroblasts from Parkinson’s Disease Patients
Source: Biomedicines. 2024 Jan 25;12(2):282. doi: 10.3390/biomedicines12020282 (PMC10886641; doi:10.3390/biomedicines12020282)
Supplement: Supplementary file 1 [file biomedicines-12-00282-s001.zip › biomedicines-2759051-supplementary.pdf]

S1. ANCOVA

A two-factor analysis of covariance (ANCOVA) was conducted to evaluate the influence of age and gender on the dependent variables [*Mitochondrial_footprint, Mitochondrial_branch_length, Individuals/mitonetworks_ratio, Invividual mitochondrial_ length, Average_mitochondrial_junction_per_cell*], with baseline scores included as a covariate. Fibroblast's donors were categorized by age (over 60 years and under 60 years) and gender (male and female). Preliminary checks validated no violation of the assumptions of normality, linearity, homogeneity of variance and regression slopes, and reliable measurement of the covariate. The interaction effect between age and sex was not statistically significant.

The tables present statistical data of covariance analysis (ANCOVA). Tables provides the pairwise comparison data using Bonferroni adjustment for multiple tests. The interaction effect between age and gender was not statistically significant.

Mitochondrial_footprint

Covariate	Sum of Squares	Mean Square	F Value	P Value
sex	30004.3	30004.3	0.16	0.68
age	30004.3	30004.3	0.16	0.68
Interaction	30004.3	30004.3	0.16	0.68

Mitochondrial_branch_length

Covariate	Sum of Squares	Mean Square	F Value	P Value
sex	0.097	0.09	0.37	0.53
age	0.097	0.09	0.37	0.53
Interaction	0.097	0.09	0.37	0.53

Individuals/mitonetworks_ratio

Covariate	Sum of Squares	Mean Square	F Value	P Value
sex	0.94	0.94	0.23	0.63
age	0.94	0.94	0.23	0.63
Interaction	0.94	0.94	0.23	0.63

Invividual mitochondrial_ length

Covariate	Sum of Squares	Mean Square	F Value	P Value
sex	0.01	0.01	0.14	0.7
age	0.01	0.01	0.14	0.7
Interaction	0.01	0.01	0.14	0.7

Average_mitochondrial_junction_per_cell

Covariate	Sum of Squares	Mean Square	F Value	P Value
sex	124.5	124.5	1.32	0.25
age	124.5	124.5	1.32	0.25
Interaction	124.5	124.5	1.32	0.25

S2.

Table S1. *Summary table.*

Line and Abbreviation	Mitochon-drial footprint	Median MN branch length	Individual mitochon-dria/MN ratio	Median individual mitochon-dria length	Average MN junctions per cell	Mitochon-drial potential	Cytosolic ROS production rate	Mitochon-drial ROS production rate
**Control1 (C1)**	–	–	–	–	–	with C2	with C2	N/A
**Control1 +H2O2 (C1S)**	–	↓ rel C1	–	↓ rel C1	↓ rel C1	with C2S	N/A	with C2S
**Control2 (C2)**	–	–	–	–	–	with C1	with C1	N/A
**Control2 +H2O2 (C2S)**	↓ rel C1S	↓ rel C1S	–	–	↓ rel C1S	with C1S	N/A	with C1S
**LRRK (L)**	–	–	–	–	–	–	–	N/A
**LRRK+H2O2 (LS)**	–	↓ rel L	–	–	↓ rel L	↓ rel C1S+C2S	N/A	–
**PINK (P)**	–	–	↑ rel C1,C2	↓ rel C1,C2	–	–	↑ rel C1+C2	N/A
**PINK+H2O2 (PS)**	–	↓ rel P	↓ rel P	–	↑ rel C1,C2	↓ rel P	N/A	↑ rel C1S+C2S
**PINK/Parkin (PP)**	↑ rel C1,C2	↑ rel C1,C2	↑ rel C1,C2	–	↑ rel C1,C2	↑ rel C1+C2	↑ rel C1+C2	N/A
**PINK/Parkin +H2O2 (PPS)**	↑ rel C1S,C2S	↓ rel PP ↑ rel C1S,C2S	↑ rel C1S,C2S	↓ rel PP	↑ rel C1S,C2S	↓ rel PP ↓ rel C1S+C2S	N/A	–
**A53T (A)**	–	↑ rel C2	–	↑ rel C1	–	↑ rel C1+C2	↑ rel C1+C2	N/A
**A53T+H2O2 (AS)**	↑ rel C2S	↑ rel C1S,C2S	–	↑ rel C1S	–	–	N/A	↑ rel C1S+C2S

A significant decrease (↓) or increase (↑) in the parameter. Relatively (rel). Parameter was not measured (N/A).

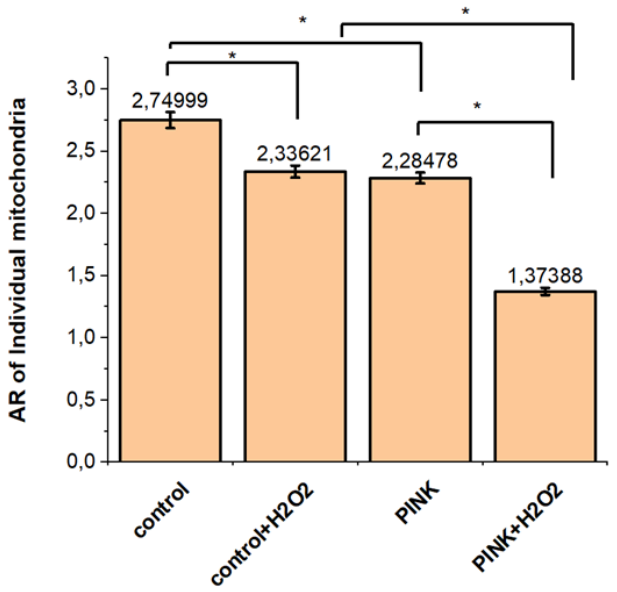

Figure S1. *Aspect ratio (AR) of individual mitochondria in Control fibroblasts and fibroblasts with PINK1 mutation measured using "Particle Analyzer" Fiji plugin.*
